# Inter-annual variability of transparent exopolymer particles in the Arctic Ocean reveals high sensitivity to ecosystem changes

**DOI:** 10.1038/s41598-017-04106-9

**Published:** 2017-06-23

**Authors:** Anja Engel, Judith Piontek, Katja Metfies, Sonja Endres, Pim Sprong, Ilka Peeken, Steffi Gäbler-Schwarz, Eva-Maria Nöthig

**Affiliations:** 1 GEOMAR Helmholtz Centre for Ocean Research Kiel, Düsternbrooker Weg 20, D-24105 Kiel, Germany; 2 Alfred Wegener Institute Helmholtz Centre for Polar and Marine Research (AWI), Am Handelshafen 12, D-27570 Bremerhaven, Germany; 3 Helmholtz Institute for Functional Marine Biodiversity, Postfach 2503, D-26111 Oldenburg, Germany

## Abstract

Transparent exopolymer particles (TEP) are a class of marine gel particles and important links between surface ocean biology and atmospheric processes. Derived from marine microorganisms, these particles can facilitate the biological pumping of carbon dioxide to the deep sea, or act as cloud condensation and ice nucleation particles in the atmosphere. Yet, environmental controls on TEP abundance in the ocean are poorly known. Here, we investigated some of these controls during the first multiyear time-series on TEP abundance for the Fram Strait, the Atlantic gateway to the Central Arctic Ocean. Data collected at the Long-Term Ecological Research observatory HAUSGARTEN during 2009 to 2014 indicate a strong biological control with highest abundance co-occurring with the prymnesiophyte *Phaeocystis pouchetii*. Higher occurrence of *P*. *pouchetii* in the Arctic Ocean has previously been related to northward advection of warmer Atlantic waters, which is expected to increase in the future. Our study highlights the role of plankton key species in driving climate relevant processes; thus, changes in plankton distribution need to be accounted for when estimating the ocean’s biogeochemical response to global change.

## Introduction

The Arctic Ocean is of key importance in the regulation of global climate and at the same time is greatly affected by climate change^1^. Arctic temperatures have increased more strongly than the global average during the recent past^2^, leading to a loss of multiyear sea-ice, which is replaced to some extent by thinner first-year ice. Due to increases in ice melting and the uptake of anthropogenic CO_2_, warming and acidification in the Arctic Ocean are expected to proceed faster than the global average^2–4^. As a consequence, productivity and element cycling in Arctic Ocean ecosystems will be altered; as sea-ice melting starts earlier in the year, a loss of ice-algae habitat occurs before solar radiation allows for algal productivity and growth^5^. Warming may also affect the vertical supply of nutrients to the euphotic zone as the upper water-column will become more stratified^6^. On the other hand, a longer ice-free period and thinner sea-ice increases underwater light availability and therefore annual phytoplankton production^7^, which may additionally be stimulated by increasing amounts of anthropogenic CO_2_^8, 9^. The overall impact of sea-ice retreat on primary production in the Arctic Ocean is still under debate^6^.

Primary production is the main source of extracellular organic polymers in seawater; a fraction of these compounds spontaneously assembles into discrete hydrogels, stabilized by divalent cation bridging^10^, and coagulates further into larger aggregates^11^. About 70 Gt C, equivalent to 10% of the global oceanic pool of dissolved organic carbon is estimated to be contained in hydrogels^12^. Assembly, aggregation, disentangling and disaggregation as well as degradation processes lead to a dynamic continuum of organic polymer gels from colloidal (~1 nm) and submicron sized gels to macrogels^13, 14^. Transparent exopolymer particles (TEP) are organic polymer gels that can be retrained on a 0.2 or 0.4 µm filter and are determined by staining with Alcian Blue, a cationic copper phthalocyanine dye that complexes COO^−^ and OSO^3–^ reactive groups of heteropolysaccharides^15^. TEP predominantly contain heteropolysaccharides, which form the hydrogel matrix due to divalent cation (Ca^2+^, Mg^2+^) and half-ester sulfate bridging between the individual molecules^14, 15^. TEP are the dominant and most extensively studied class of discrete gel particles in marine systems^15^, and were shown to be involved in a variety of ecological and biogeochemical processes. Being readily abundant and adhesive, TEP play a pivotal role in particle aggregation processes and carbon export fluxes^16^. Variations in carbon export fluxes influenced by TEP are assumed to be significant for CO_2_ sequestration on a global scale^17, 18^. According to a global ocean carbon cycling model, a 5% increase in TEP export between preindustrial and present day times could be responsible for an increase in CO_2_ sequestration by 97 Gt C for the time period 1770–2200^18^.

Via sea-spray, colloidal and submicron sized polysaccharide gels are emitted to the atmosphere, where they serve as nuclei for vapor condensation, capable of inducing low level cloud and ice crystal formation^19, 20^. For the Arctic, a direct link between climate processes and polysaccharide gels as precursors of cloud condensation nuclei (CCN) and ice nucleating particles (INP) has been suggested^19–21^. A higher biogenic CCN contribution above the Arctic could therefore alter the radiative forcing in this area and counteract the effect of climate change.

**Figure 1:**
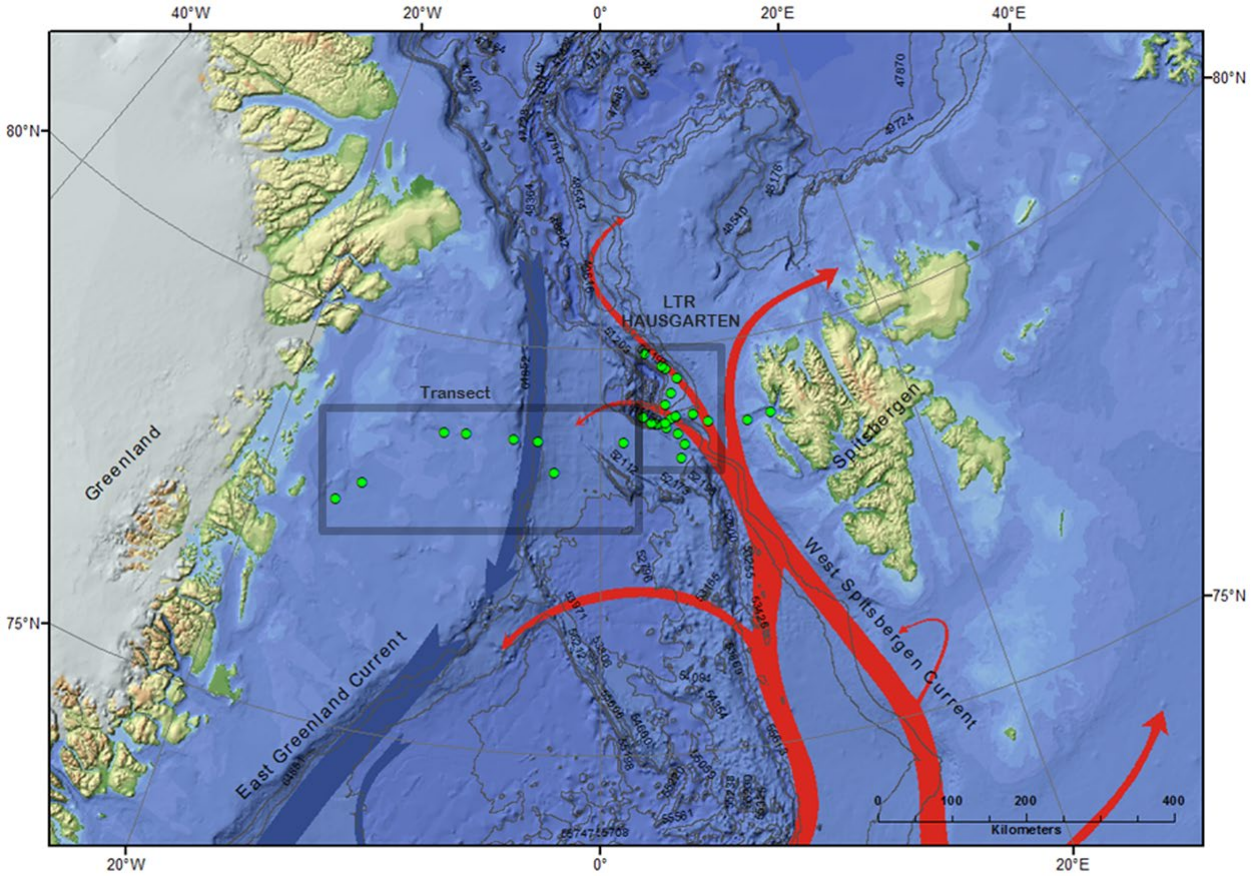
Map of the study area in the Fram Strait (Arctic Ocean), with the LTER (Long-Term Ecological Research) HAUSGARTEN observatory visited in 2009–2012 and 2014, as well as the transect stations sampled in 2014. The map was created using ArcGIS 10.3 and based on the General Bathymetric Chart of the Oceans (GEBCO)-08 grid, version 20100927, http://www.gebco.net, with permission from the British Oceanographic Data Centre (BODC).

Estimating the importance of marine gels, specifically of TEP, for particle export and climate control in the Arctic, however, is impeded by a lack of quantitative data. Here, we show for the first time temporal and spatial variability of TEP abundance in the Fram Strait, the Atlantic gateway to the Central Arctic Ocean. We identify relationships between TEP occurrence and ecosystem changes including chlorophyll *a* (Chl *a*) concentration, phytoplankton abundance and microbial community composition. In every summer (June/July) of the years 2009, 2010, 2011, 2012, and 2014 field samples were collected between the sea surface and 150 m depth. Samples were taken from between 12 and 25 stations at the Long-Term Ecological Research (LTER) observatory HAUSGARTEN (78. 42°N, 2.27°E and 79.50°N, 6.20°E) with the research vessel POLARSTERN (ARKXXIV/2 in 2009), (ARKXXV/2 in 2010), (ARKXXVI/2 in 2011), (ARKXXVII/2 in 2012), and (ARKXXVIII/2, PS85 in 2014) (Fig. 1, Table S1). In 2014, in addition to the HAUSGARTEN stations, a longitudinal transect with 13 stations was sampled across the Fram Strait along ~78.5°N, and between 15.5°W and 6.10°E.

Water temperatures between 2009 and 2014 varied between −1.80 and 7.74 °C, salinity between 30.15 and 35.53 depending on ice melt and meandering of the West Spitsbergen (WSC) current carrying North-Atlantic waters poleward and the East Greenland current (EGC) transporting polar waters to the South (Fig. 1, Table S1). Overall, seawater temperature in our data set was positively related to salinity (r² = 0.40, n = 436) reflecting the northeast-bound inflow of more saline and warmer Atlantic water.

**Figure 2:**
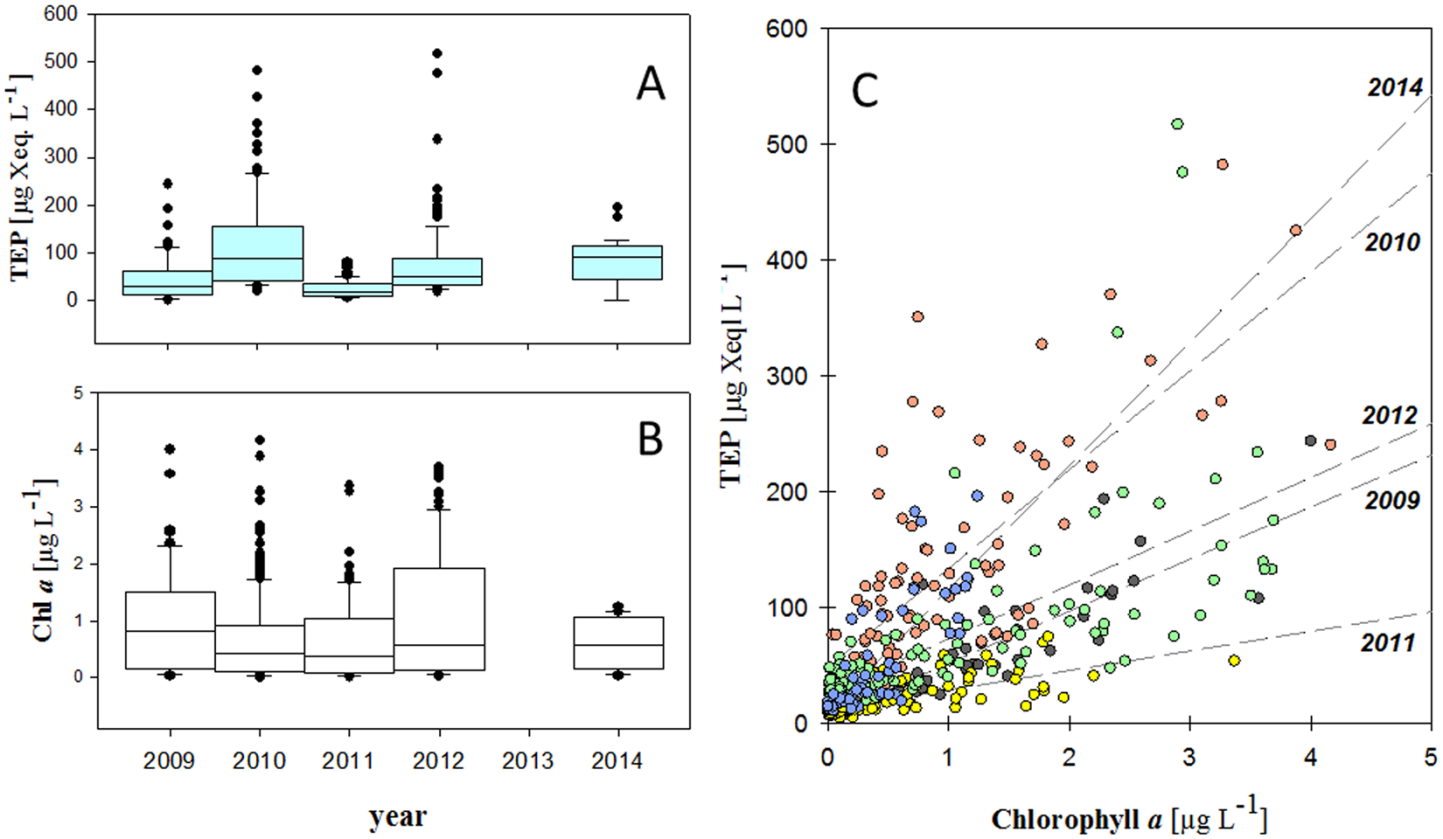
(a–c) Variability of transparent exopolymer particles (TEP, **A**) and Chlorophyll a (Chl *a*, **B**) concentrations at the HAUSGARTEN Arctic monitoring site during summer (2009–2014). TEP and Chl *a* concentrations were significantly correlated in each year (*p* < 0.001), but the increase in TEP relative to Chl *a* concentration varied strongly between years (**C**). Color code (**C**): yellow 2011, grey 2009, green 2012, red 2010, blue 2014.

**Figure 3:**
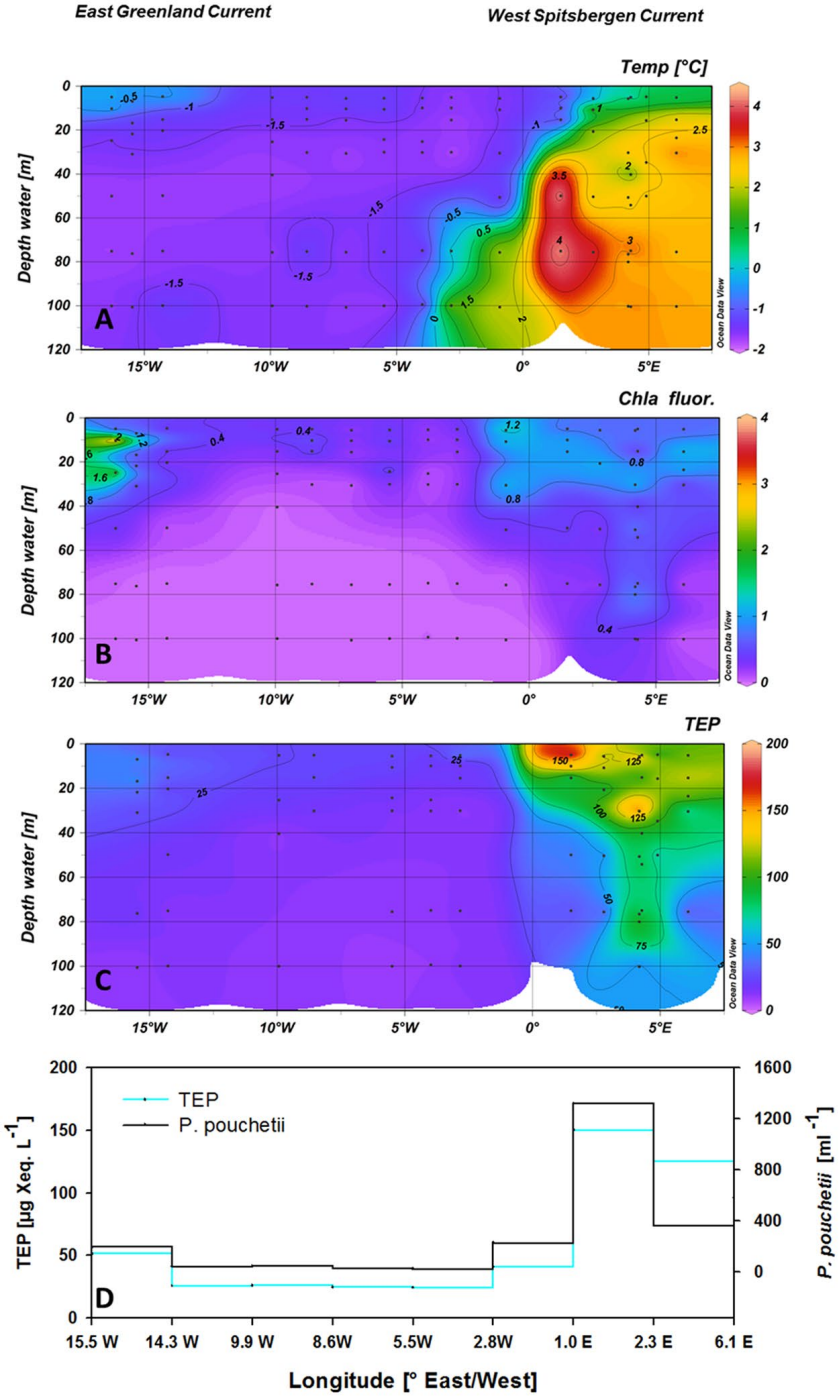
(a–d) Depth distribution of temperature (°C; **A**), (b) TEP concentration [µg Xeq. L^−1^; (**B**)] and Chl *a* concentration [µg L^−1^; (**C**)] as well as abundance of *Phaeocystis pouchetii* and TEP concentration at the depth of the Chl *a* maximum across the Fram Strait along the 78.5°N transect in 2014 (**D**). Figure 3(A–C) were created using Ocean Data View 4^52^.

TEP concentrations at the time series stations ranged between 5 and 517 µg Xanthan Equivalents (Xeq.) L^−1^ (average 75 ± 78 µg Xeq. L^−1^, *n* = 362) and were highest in or above the Chl *a* maximum. Assuming a carbon content of 63% (w/w)^22^ this suggest an organic carbon contribution by TEP in the order of 10^0^–10^2^ µg C L^−1^. TEP abundance was significantly related to Chl *a* concentration in each year (*p* < 0.001), but high inter-annual variability in the response ratio of TEP to Chl *a* production (Δ[TEP]:Δ[Chl *a*]) was observed (Fig. 2a–c, Table S1). Hence, no overall general relationship between Chl and TEP was observed, suggesting that Chl *a* concentration alone is not a good predictor for TEP abundance in the Arctic. Highest TEP concentrations were observed in 2010 when Δ[TEP]:Δ[Chl *a*] was about five times higher than in the following year 2011 (Fig. 2c, Table S1). Pronounced differences in TEP distribution were observed along the ~78.5°N-transect in 2014, with highest TEP concentration in the warmer Atlantic waters of the WSC where phytoplankton biomass was dominated by the prymnesiophyte *Phaeocystis pouchetii* (Fig. 3a–d, Table S2). *P*. *pouchetii* is a boreal phytoplankton species that produces toxins, which are potentially harmful to fish larvae after ingestion^23^. It occasionally forms massive blooms in the sub-arctic North Atlantic and Fram Strait^24^. Earlier observations from LTER HAUSGARTEN indicated an increasing contribution of *P*. *pouchetii* to phytoplankton abundance in surface waters from 1997–2010 along with higher seawater temperatures and generally increased autotrophic biomass^24–26^. Anomalies of higher seawater temperature at this site have been related to an intensification of warmer North-Atlantic waters flowing polewards^27, 28^.

**Figure 4:**
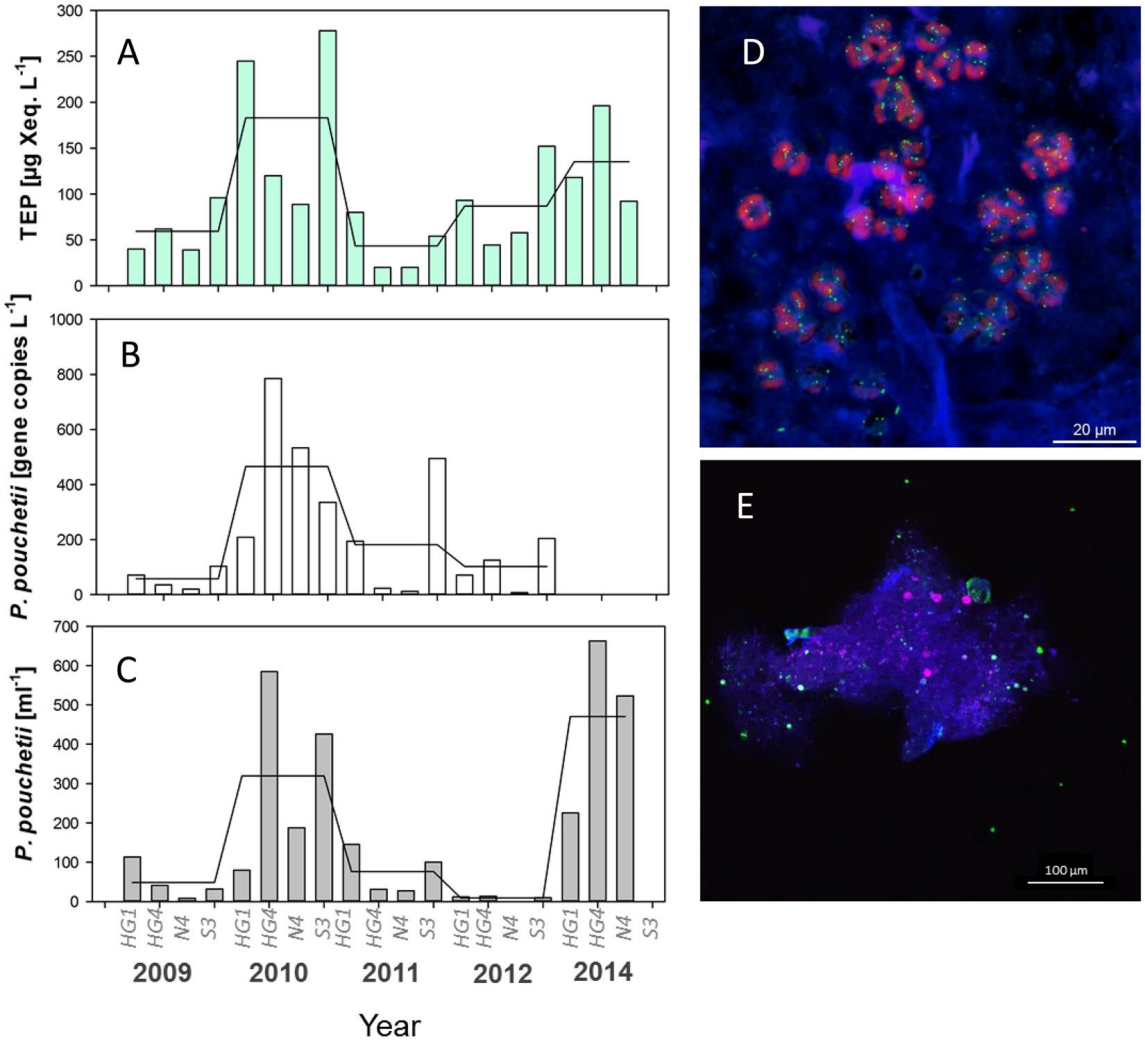
Concentration of TEP [(**A**); µg Xeq. L^−1^] and abundance of *P*. *pouchetii* as determined by 454 sequencing [(**B**); sequence abundance in this analysis] and q-PCR analyses [(**C**); ml^−1^] at four time series stations from 2009 to 2014. Association of polysaccharide gels (TEP) with *P*. *pouchetii* (**D**) and within sinking particle aggregates (**E**) as determined by confocal laser scanning microscopy (CLSM) using a conjugate of Concanavalin A and Alexa Fluor 633 to stain polysaccharide gels (blue), SYTO 9 to stain bacteria (green) and Chl *a* autofluorescence (red). Pictures by Jan Michels﻿ (D) and Kathrin Busch (E).

To better understand variability of TEP abundance in the Fram Strait, we analysed data from the Chl *a* maximum of four time series stations (HG1, HG4, N4, S3) during the period 2009–2014 and from the transect across the Fram Strait in 2014 and related TEP abundance to (1) plankton community composition determined by 454-pyrosequencing of 18SrDNA (Figure S1), (2) to phytoplankton abundance determined by microscopy (S3) and (3) to *P*. *pouchetii* abundance determined by real-time quantitative PCR (q-PCR). A strong relationship was determined between abundances of TEP and *P*. *pouchetii*, both spatially, along the 78.5°N transect (*p* = 0.002, *n* = 9, Fig. 3d) and temporally between 2009 and 2014 (Fig. 4a–c) (p = 0.005, *n* = 19). 2010 and 2014, the years with highest *P*. *pouchetii* dominance, showed three- to fivefold higher TEP concentration than years when plankton community was dominated by other species (Figure S1). Moreover, the highest ratio of TEP to Chl *a* concentration was observed in these years (Fig. 3c, Table S1), suggesting that TEP production by *P*. *pouchetii* exceeds those of other phytoplankton species.

Species of *Phaeocystis* are well known to produce and release copious amounts of neutral and acidic polysaccharides^29, 30^. Intracellular glucans serve as storage polysaccharides, while extracellular polysaccharides released from exponentially growing cells and senescent colonies can induce TEP formation. A close coupling between TEP abundance and particulate organic matter export in the course of *P*. *pouchetii* blooms was observed previously for Arctic and sub-Arctic systems, suggesting that aggregation with TEP is the main driver of episodic mass sedimentation^31–33^. Moreover, high particle export fluxes in the Arctic Ocean have been related to the occurrence of *P*. *pouchetii* blooms^34^. Also for the Southern Ocean, aggregation of TEP with phytoplankton was suggested to enhance particle export fluxes^35^.

For our samples from the HAUSGARTEN, microscopic inspection confirmed that TEP were readily abundant in samples with *P*. *pouchetii* and formed the matrix of sinking particle aggregates (Fig. 4d,e), corroborating the importance of TEP in particle aggregate formation and carbon export fluxes.

However, the efficiency of the biological carbon pump for sequestration of atmospheric CO_2_ also depends on the attenuation of carbon flux with depth. For the Arctic Ocean, it has been suggested that carbon export attributed to *P*. *pouchettii* diminishes quickly at shallow depths (~100 m) and may not contribute to CO_2_ storage on longer time scales^31^. Since TEP are almost neutrally buoyant, a higher proportion of TEP reduces the settling velocity of aggregates^36^ and may favour aggregate decomposition at shallower depth. Still unknown is the direct effect of receding sea-ice on export processes. Export of a *P*. *pouchetii* bloom well below 100 m was observed at the sea-ice edge, with remineralization rates in the mesopelagic zone being comparable to diatom derived material^34^. However, deep export of aggregates of *P*. *pouchettii* with TEP likely relies on the presence of heavier particles. A community shift from diatoms that can act as bio-mineral ballast, to *P*. *pouchetti* may therefore lead to a shallowing of the carbon remineralisation depth, potentially allowing for CO_2_ exchange with the atmosphere after wintertime convection. Moreover, TEP are carbohydrate-rich substrates for heterotrophic microorganisms. It has been suggested earlier that an increased supply with carbohydrates may stimulate the metabolism of Arctic bacteria, leading to enhanced respiration and higher loss of CO_2_ to the atmosphere^37^.

Besides favourable growth conditions, such as nutrients, temperature and light, the spatial distribution of phytoplankton species is largely determined by ocean circulation. Intensified northward advection of Atlantic water is one consequence of climate change and is expected to have profound effects on Arctic ecosystem structures^1^. So far, changes in plankton community composition are rarely included when predicting future biogeochemical cycling in the ocean. Our results suggest that changes in the abundance of phytoplankton key species like *P*. *pouchetii* contribute to variability of biogeochemical components and may superimpose or even amplify direct effects of global change. For the Arctic Ocean, a shift from a community dominated by diatoms in the past to an enhanced abundance of *P*. *pouchetii* as a consequence of changes in Atlantic inflow^24^ may greatly intensify TEP production, with potential implications for carbon cycling, food-web structure and air-sea interactions as mentioned above. Yet, how ecological and biogeochemical processes in the Arctic Ocean and elsewhere will evolve in the future is difficult to predict. Simultaneous changes in an organism’s chemical and physical environment may either amplify or dampen the organism’s response to each individual change, and also induce complex ecological responses with potentially cascading effects^38^. Prevailing species may adapt physiologically to their changing environment or be replaced by more adapted ones.

During the last decades, immense scientific effort has been directed towards better understanding and predicting responses of marine organisms and communities to the consequences of global change. Sensitivities to short-term changes in temperature, sea-ice extent, *p*CO_2_ and pH, or nutrient loads have been observed for selected plankton species and communities in experimental simulations^8, 9, 38, 39^. Ocean acidification may increase extracellular release and TEP formation by up to 18–33%^8, 39^, respectively, which is rather small compared to 84–500% higher TEP concentrations observed during years of *P*. *pouchetii* dominance in this study. Our results suggest that changes in phytoplankton community structure leave marked footprints on TEP abundance in the ocean.

Understanding how climate-induced changes in ocean circulation patterns will redistribute plankton species and alter plankton community composition may therefore be necessary to accurately predict future biogeochemical cycling. In this respect, time series observations are an invaluable tool as they monitor the integrated response of an ecosystem together with changes in the physical and chemical environment.

## Methods

### Field sampling

A SEA-BIRD CTD system, equipped with a 24 Niskin bottle (12 L) rosette sampler, was used to determine depth profiles of temperature and salinity, and to collect seawater. At each station, water was collected at several depths between 5 and 150 m. All samples were processed on board immediately after sampling. Hydrographic data for this period including seawater temperature and salinity were retrieved at PANGAEA (doi:10.1594/PANGAEA.753658, 754250, 78140, 800427, 837425).

### Molecular analyses

Samples were taken during the upcasts at the vertical maximum of Chl *a* fluorescence determined during the downcasts. Stations investigated include the HAUSGARTEN stations HG1 (79.14°N, 6.09°W), HG4 (79.07°N, 4.18°W), N4 (79.76°N, 4.28°W), and S3 (78.61°N, 5.97°W). The depth of the Chl *a* maximun varied between 10–50 m among the different stations.  Two liter subsamples were taken in PVC bottles from the Niskins. Particulate organic matter for molecular analyses was collected by sequential filtration of one water sample through three different mesh sizes (10 µm, 3 µm, 0.4 µm) on 45 mm diameter Isopore Membrane Filters at 200 mbar using a Millipore Sterifil filtration system (Millipore, USA). Phytoplankton community structure in samples was characterized using 454 sequencing and RT-PCR analyses.

### Chlorophyll a (Chl *a*)

The concentration of chlorophyll a (Chl *a*) was determined from 0.5 – 2 L of seawater filtered onto glass fibre filters (Whatman GF/F) under low vacuum (<200 mbar); the filters were stored at −20 °C before analysis. Pigments were extracted with 10 ml of 90% acetone. The filters were treated with an ultrasonic device in an ice bath for less than a minute, and then further extracted in the refrigerator for 2 h. Subsequently they were centrifuged for 10 minutes at 5000 rpm at 4 °C prior to measurement. Chl *a* concentration was determined fluorimetrically (Turner Designs), together with total phaeophytin concentration after acidification (HCl, 0.1 N) slightly modified to the methods described in Edler (1979)^40^ and Evans *et al*. (1980)^41^, respectively. The standard deviation of replicate test samples was <10%.

### Transparent exopolymer particles (TEP)

TEP were determined after Passow and Alldredge (1995)^42^. Briefly, 5- 500-mL samples were filtered onto 0.4 µm Nuclepore filters, stained with 1 mL of an aqueous solution of Alcian Blue (0.02% w/w at pH 2.5) and rinsed with distilled water. Filters were kept frozen until analysis, which was performed within 4 months of collection. Each filter was soaked for at least two hours with 6 mL of 80% H_2_SO_4_ in order to dissolve the particulate matter, and absorption of the solution was measured at 787 nm in a 1-cm cuvette. The acidic polysaccharide Gum Xanthan was used as a standard to relate Alcian Blue adsorption to TEP. The calibration factor (*f*) was determined for every sampling campaign, yielding a factor of *f* = 41 for all samples from 2009 to 2012 and *f* = 140 for samples from 2014 (ARK28). Triplicates, occasionally duplicates, were analysed for each sample. The detection limit of the measurements was 5 µg Gum Xanthan equivalents (Xeq.) L^−1^ and the standard deviation of triplicate samples was <10% in most cases.

### Microscopy

In order to study the species composition of unicellular plankton organisms present at the sampling sites, water samples were collected from the chlorophyll a maximum layer and cell abundances counted. Samples were stored cool in dark brown glass bottles and preserved with hexamine-neutralized formaldehyde (0.5–1% final concentration) until further analyses. In the laboratory, quantitative microscopic analysis of phytoplankton was conducted using 50 mL aliquots. Cells were allowed to settle for at least 48 h before being identified and counted with an inverted microscope at 100 × , 200 × and 400 × magnification. Some species, such as very small flagellates, were identified only to genera. Phytoplankton species were grouped into the following functional groups: diatoms, nanoflagellates, autotrophic dinoflagellates, coccolithophores and *P*. *pouchetii*. Phytoplankton carbon (PPC) was calculated by measuring the linear dimensions of cells in order to estimate cell volume. Conversion to carbon assumed a carbon to plasma volume ratio of 0.13 pg Cµm^−3^ for thecate dinoflagellates and 0.11 pg Cµm^−3^ for all other taxonomic groups^40^. *Phaeocystis pouchetii* carbon was calculated only for the cells; no correction was made for the gelatinous membrane. Therefore PPC values given here for *P*. *pouchetii* are slightly underestimated.

For confocal laser scanning microscopy (CLSM) samples were filtered under low vacuum (<200 mbar) onto 25 mm black polycarbonate filters with a pore size of 0.2 μm and stored at −20 °C until further analysis. For analyses, filters were stained for 20 min in the dark at room temperature with sodium bicarbonate buffer (concentration: 0.1 mol L^−1^) containing SYTO 9 (concentration: 5 µmol L^−1^) and a conjugate of the lectin Concanavalin A (Con A) and the fluorescent dye Alexa Fluor 633 (concentration: 0.1 mg mL^−1^). Fluorescence of particles was examined with a Leica TCS SP5 II CLSM system with a Leica HC PL APO 20×/0.75 IMM CS2 objective. The following wavelengths were used to excite and detect the different fluorescence: SYTO 9: 488 nm excitation, 495–555 nm emission; Alexa Fluor 633: 633 nm excitation, 640–680 nm emission; chlorophyll autofluorescence: 488 nm excitation, 640–740 nm emission.

### DNA isolation

Genomic DNA was extracted from cells collected from all filters after sequential filtration. DNA extraction was carried out using the Nucleospin Plant II kit (Machery-Nagel, Düren, Germany) following the manufacturer’s protocol. Genomic DNA was eluted in 60 µL elution buffer provided by the manufacturer. The extracts were stored at −20 °C until analysis.

### PCR-amplification of 18S rDNA

For 454-pyrosequencing, a ~670 bp fragment of the 18 S rDNA containing the hypervariable V4 region was amplified separately from each filter fraction with the primer set 528 F (GCG GTA ATT CCA GCT CCA A) and 1055 R (ACG GCC ATG CAC CAC CAC CCA T)^43^. The 18 S rDNA was amplified separately from the different fractions to minimize the PCR-bias related to variability in amplification efficiency of differently sized taxa. All PCRs had a final volume of 50 µL and contained: 0.02 U HotMaster Taq polymerase (5′Prime); the 10-fold polymerase buffer according to manufacturer’s specification; 0.4 mg/mL BSA; 0.8 mM (each) dNTP (Eppendorf, Germany); 0.2 µM each primer and 1 µL of template DNA. PCR amplification was performed in a thermal cycler (Eppendorf, Germany) with an initial denaturation (94 °C, 5 min) followed by 35 cycles of denaturation (94 °C, 1 min), annealing (58 °C, 2 min), and extension (72 °C, 2 min) with a single final extension (72 °C, 10 min). The PCR products were purified with a Mini Elute PCR Purification kit (Qiagen, Germany). Subsequently, equal volumes of PCR-products were pooled and subjected to 454-pyrosequencing. Finally, the sequencing of the amplicon was performed by GATC Biotech (Germany), using a 454 GS FLX Titanium sequencer (Roche, Germany) for the following samples: (ARKXXIV/2) (HG1, HG4, S3, N4), (ARKXXV/2) (HG1, HG4, S3), (ARKXXVI/2) (HG1, HG4, S3, N4), (ARKXXVII/2) (HG1, HG4, S3, N4). ARKXXV/2 (N4) was sequenced by LGC Genomics GmbH (Berlin, Germany). Sequences generated in this study have been deposited at the European Nucleotide Archive (ENA) under Accession PRJEB21238.

### Data analysis

Raw sequence reads were processed using the analysis pipeline Quantitative Insights Into Microbial Ecology Version 1.8.0 (QIIME)1. The primer set used in this study amplifies a PCR product of ~500 bp including the V4-region of the 18 S rRNA gene. The forward primer 528 F, used for the sequencing, attaches approximately 25 bp upstream of the V4 region, which has an approximate length of 230 bp^44^. Thus, sequence reads with a length under 250 bp were excluded from further analysis to ensure including the complete V4 region in the analysis and to omit short reads. The quality score was set to 25 and eight homopolymers and two primer mismatches were allowed. Chimeric sequences in the remaining data set were eliminated from further analyses based on an assessment using the software UCHIME^45^ within QIIME^46^. The resulting high quality reads were subsampled to allow comparison of sequence abundance in the different samples. Subsequently these high quality sequences were grouped into operational taxonomic units (OTUs) at the 97% similarity level using UCLUST^47^. The 97% similarity level has shown to be the most suitable to reproduce original eukaryotic diversity^48^ and also has the effect of bracing most sequencing errors^49^. Furthermore, known intragenomic SSU polymorphism levels can vary by 2.9% in dinoflagellate species^50^. OTUs composed of less than 4 sequence reads were removed from the analysis. The remaining sequences were aligned using the SILVA reference database (SSU Ref 119). Unassigned OTUs and those assigned to *Ophistakonta* were excluded from further analysis in a final cleaning step.

### Quantitative PCR-assay

Quantitative PCR was carried out in a nested 2-step approach. In the first step total eukaryotic 18 S rDNA was amplified using the universal primer-set 1F-(AACTGGTTGATCCTGCCAGT)/1528R-(TGATCCTTCTGCAGGTTCA-CCTAC) modified after a primer set published previously^51^.

PCR-amplifications were performed in a 20 µL volume in a thermal cycler (Eppendorf, Germany) using 1x HotMasterTaq buffer containing Mg2+, 2.5 mM (5′Prime); 0.5 U HotMaster Taq polymerase (5′Prime, Germany); 0.4 mg/mL BSA; 0.8 mM (each) dNTP (Eppendorf, Germany); 0.2 µM of each primer (10 pmol/µL) and 1 µL of template DNA (genomic DNA field samples). The amplification was based on 35 cycles, consisting of 94 °C for 1 min, 54 °C for 2 min and 72 °C for 2 min, followed by 1 min denaturation at 94 °C and finished by a final extension of 10 min at 72 °C. Subsequently PCR products were purified using a QIAquick PCR purification kit (Qiagen, Hilden, Germany). In the second step a qPCR-assay was carried out using a species specific primer-set 82 F (GTGAAACTGCGAATGGCTCAT)/P1np (CGGGCGGACCCGA-GATGGTT) for *Phaeocystis pouchetii*. The quantitative PCR-assays were performed on a 20 µL sample using a 7500 Fast Real-Time PCR-System (Life Technologies Corporation; Applied Biosystems, USA) thermal cycler (Eppendorf, Germany) using 1x SYBR Select Mastermix (Life Technologies, USA); 0.2 µM of each primer (10 pmol/µL) and 2.5 µL of the purified 18 S rDNA PCR-fragment. The amplification was based on 40 cycles, consisting of 95 °C for 10 min, 95 °C for 15 sec, 66 °C for 1 min. The quantitative PCR assay was calibrated with a dilution series of a laboratory culture of *Phaeocystis pouchetii*. Based on this calibration, CT values were transformed into cell numbers using the following equation: CT = −2.123 ln (cell numbers) +38.788.

## Electronic supplementary material

Supplementary Information
