# Supplementary material for: Inter-annual variability of transparent exopolymer particles in the Arctic Ocean reveals high sensitivity to ecosystem changes
Source: Sci Rep. 2017 Jun 23;7:4129. doi: 10.1038/s41598-017-04106-9 (PMC5482855; doi:10.1038/s41598-017-04106-9)
Supplement: Supplementary file 1 — Supplementary Information [file 41598_2017_4106_MOESM1_ESM.pdf]

Supplementary information to: Inter-annual variability of transparent exopolymer particles in the Arctic Ocean reveals high sensitivity to ecosystem changes

Anja Engel[1], Judith Piontek[1], Katja Metfies[2,3], Sonja Endres[1], Pim Sprong[2], Ilka Peeken[2], Steffi Gäbler- Schwarz[2] and Eva-Maria Nöthig[2]

[1] GEOMAR Helmholtz Centre for Ocean Research Kiel (GEOMAR), Düsternbrooker Weg 20, D-24105 Kiel, Germany

[2] Alfred Wegener Institute Helmholtz Centre for Polar and Marine Research (AWI) - Am Handelshafen 12, D-27570 Bremerhaven, Germany

[3] Helmholtz Institute for Functional Marine Biodiversity, Postfach 2503, D-26111 Oldenburg, Germany

*Corresponding author: aengel@geomar.de

Supplemental information

Table S1: Position of stations visited during the Arctic field campaigns to sample for TEP concentration.

Station	HG1	HG2	HG3	HG4	HG5	HG6	HG7	HG8	HG9	S1	S2	S3
°North	79.14	79.13	79.11	79.01	79.06	79.06	79.06	79.06	79.13	78.92	78.78	78.61
°West	6.11	4.94	4.60	4.33	43.65	3.55	3.48	3.33	2.84	5.00	5.33	5.07
Station	N1	N2	N3	N4	N5	411	424	426	429	437	444	455
°North	79.28	79.41	79.60	79.74	79.93	77.72	77.99	78.81	78.83	78.83	78.83	78.45
°West	4.33	4.70	5.17	4.50	3.18	-15.48	-14.28	-9.93	-8.56	-5.50	-3.98	-2.83

Table1 S2: Range of variables in the upper 125m of the LTR HAUSGARTEN in summer (July) from 2009-2012 and along the 78.5°N transect in 2014.

	ARK_XXIV_II	ARK_XXV_II	ARK_XXVI_II	ARK_XXVII_II	ARK_XXVIII_II
Time	10.-17.07.2009	2.-23.07.2010	14.-29.07.2011	10.-26.07.2012	14.-28.06.2014
T°C	-1.18 – 6.71	-1.76 – 6.85	-1.58 – 7.74	-1.76 – 7.74	-1.79 – 4.49
S	32.69 - 35.13	30.27 – 35.16	30.15 – 35.53	30.15 – 35.53	31.11 – 35.14
Chl a (µg L^{-1})	0 - 4.0	0 - 4.17	0 – 3.37	0 – 4.17	0.004 – 3.51
TEP (µg Xeq. L^{-1})	7.1 - 243	19 - 482	4.1 – 80	4.1 - 517	10 - 196
TEP vs. Chl a slope	45±3 (r^2=0.88, n=62)	86±8 (r^2=0.78, n=99)	17±2 (r^2=0.69, n=79)	47±35 (r^2=0.69, n=108)	107±10 (r^2=0.65, n=62)

Table S3: Phytoplankton carbon calculated at the depth of the Chl *a* maximum across the Fram Strait along the 78.5°N transect in 2014.

Longitude W° -> E°	– 15,5	– 14,3	– 9,9	– 8,6	– 5,5	– 2,8	1,01	4,28	6,1
	411	424	426	429	437	455	486	473	470
Station depth of chl.a max	20m	20m	25m	15m	25m	20m	15m	40m	23m
Phytoplankton carbon (µg/L)									
Diatoms	0.07	0.02	0.03	0.81	0.27	0.00	0.10	0.15	0.24
Nanoflagellates	0.57	0.26	0.13	0.10	0.26	0.14	0.30	1.74	4.10
Dinoflagellates	< 0.001	< 0.001	< 0.001	0.02	0.01	< 0.001	0.30	< 0.001	5.83
Phaeocystis pouchetii	0.43	1.76	0.19	0.26	0.17	0.65	0.67	1.53	1.56
Coccolithophores	< 0.001	< 0.001	< 0.001	< 0.001	< 0.001	< 0.001	0.01	< 0.001	0.26

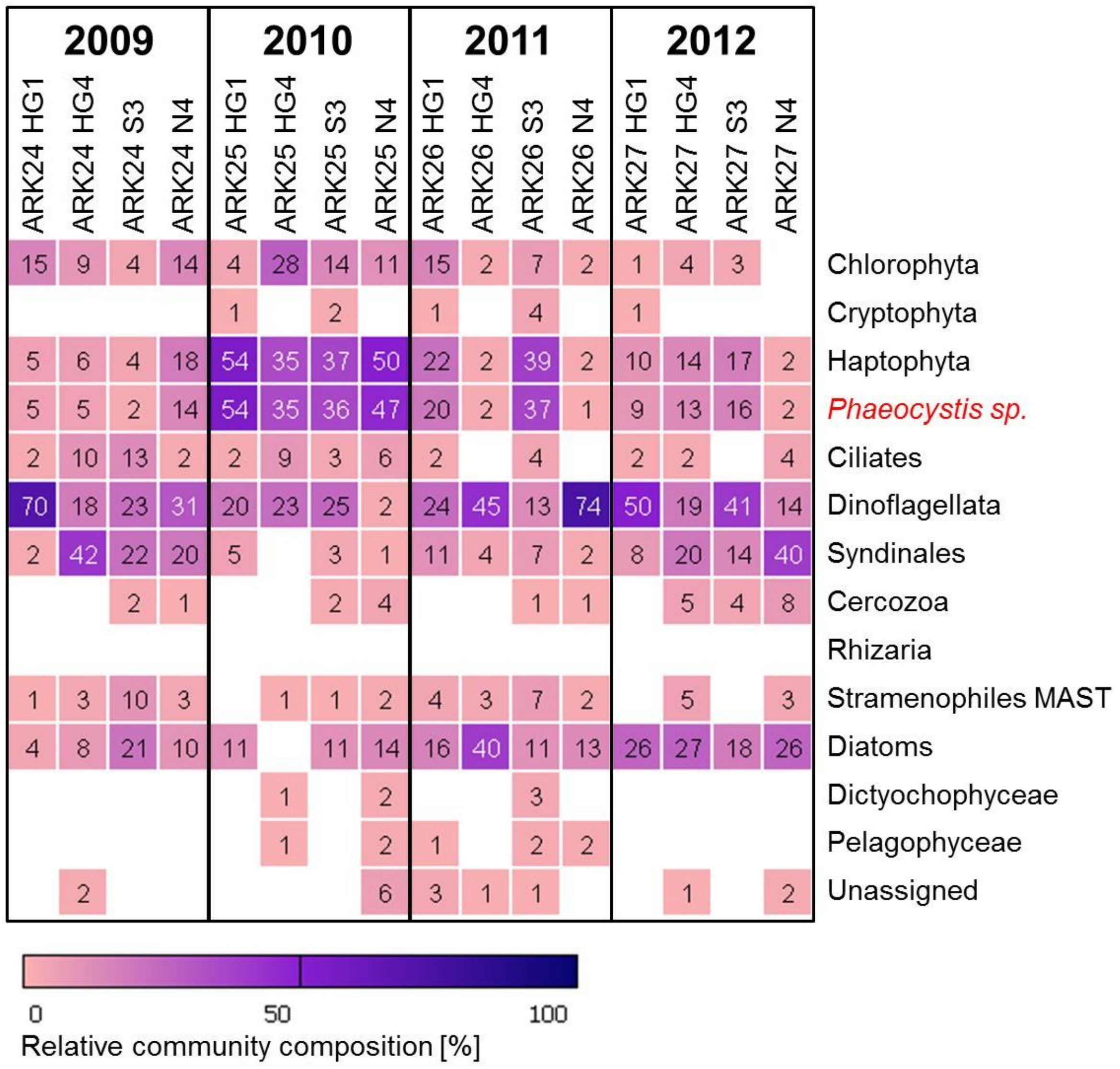

Figure S1. Relative community composition of abundant groups that constitute >1% of sequences in a sample based on 454- sequencing of 18SrDNA. Species of *Phaeocystis* dominated the group haptophyta.
